# Plasma and Polymers: Recent Progress and Trends

**DOI:** 10.3390/molecules26134091

**Published:** 2021-07-05

**Authors:** Igor Levchenko, Shuyan Xu, Oleg Baranov, Olha Bazaka, Elena P. Ivanova, Kateryna Bazaka

**Affiliations:** 1 Plasma Sources and Application Centre, National Institute of Education, Nanyang Technological University, Singapore 637616, Singapore; shuyan.xu@nie.edu.sg; 2 Faculty of Aircraft Engines, National Aerospace University, 61070 Kharkiv, Ukraine; o.baranov@khai.edu; 3 School of Science, RMIT University, P.O. Box 2476, Melbourne, VIC 3001, Australia; olga.bazaka@jcu.edu.au (O.B.); elena.ivanova@rmit.edu.au (E.P.I.); 4 School of Mechanical, Medical and Process Engineering, Queensland University of Technology, Brisbane, QLD 4000, Australia; 5 School of Engineering, The Australian National University, Canberra, ACT 2601, Australia

**Keywords:** polymers, plasma, polymer functionalization

## Abstract

Plasma-enhanced synthesis and modification of polymers is a field that continues to expand and become increasingly more sophisticated. The highly reactive processing environments afforded by the inherently dynamic nature of plasma media are often superior to ambient or thermal environments, offering substantial advantages over other processing methods. The fluxes of energy and matter toward the surface enable rapid and efficient processing, whereas the charged nature of plasma-generated particles provides a means for their control. The range of materials that can be treated by plasmas is incredibly broad, spanning pure polymers, polymer-metal, polymer-wood, polymer-nanocarbon composites, and others. In this review, we briefly outline some of the recent examples of the state-of-the-art in the plasma-based polymer treatment and functionalization techniques.

## 1. Introduction

Commonly referred to as the fourth state of matter, plasma is produced when the molecules in the gas become ionized. The resulting mixture contains various quantities of highly chemically and biologically reactive species including energetic electrons, positively and negatively charged ions as well as particles with a neutral charge and radicals and fragments that result from the breakdown and recombination of molecules [1,2]. The charged nature of species in plasmas enables one to control their movement in space, allowing for the directional and selective generation of intense fluxes of energy and matter across space. This combination of reactivity and selectivity has made plasmas a useful tool in a variety of systems, from medicine where it can be used to selectively induce apoptotic processes in cancer cells [3,4,5], to biology where it can be used to upregulate sugar metabolism and alcohol tolerance in industrial yeast or inhibit resistant bacterial biofilms [6,7,8], to agriculture and aquaculture where plasmas can enhance survival and productivity of commercially significant species of plants and fish [9,10]. In addition to changing the behavior of biological objects, plasma-enhanced technologies are used extensively in materials processing and engineering [11,12,13,14], where plasma-generated species can act as catalysts in material degradation, as building blocks in material assembly, and as modifiers of surface properties. While in material engineering the ability to control the directional flow of plasma-generated species is primarily used to deliver precise quantities of species to the surface of materials to induce desired material assembly and/or modification, in aerospace engineering [15,16,17,18,19,20,21], devices such as plasma thrusters use a combination of electric and magnetic fields to accelerate and expel plasma at high velocity, producing thrust for satellites [22,23,24]. In each of these examples, from medicine to space propulsion, the interactions that take place when plasma-generated particles and effects come into contact with matter play a critical role in defining process efficiency and selectivity of the effects that are being induced in this matter. This is because these interactions influence the nature and quantities of fluxes of matter and energy that is being extracted from the bulk of the plasma and delivered to the molecules that comprise the solid or liquid. In many of the systems used for material processing, the inherent difference in the speed with which energetic, fast, and light electrons arrive at the surface compared to heavier, slower ions leads to the formation of a layer of space above the surface termed plasma sheath. The width of this layer is determined by the specifics of the plasma and the characteristics of the surface, and hence can be controlled by tuning the properties of these two media. In turn, the properties of the sheath influence the extraction of fluxes of material and energy from the plasma bulk and their delivery to the surface, providing a simple yet efficient mechanism for control over the plasma-surface interfacial reactions across multiple temporal and morphological scales [25,26,27,28].

**Figure 1:**
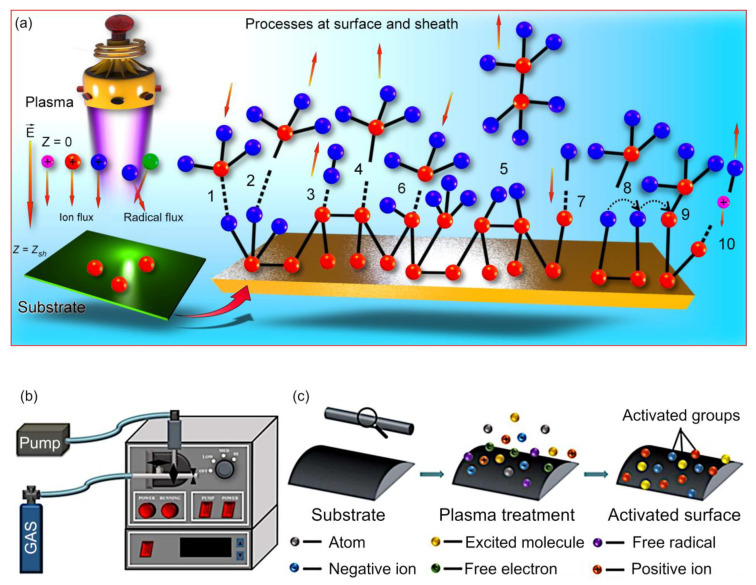
(**a**) When the surface of the substrate is exposed to plasma, multiple reactions between reactive molecules and radicals can lead to the formation of a film. In plasma, the ions and radicals are formed and accelerate toward the surface. Once they arrive at the surface of the substrate, the following reactions may take place: (1) adsorption and (2) desorption of reactive molecules; abstraction of H by (3) H radical and (4) other radicals; (5) reactive molecule abstraction by radical; direct chemisorption of (6) reactive molecules, and (7) H onto dangling bond; (8) transfer of adsorbed molecules on hydride sites; (9) chemisorption of adsorbed molecules into dangling bonds; and (10) sputtering of mono-hydride sites by ions. Reproduced Marvi et al. (2017) [29] under conditions of the BY-NC license. Diagram of the plasma treatment equipment: (**b**) plasma device; (**c**) mechanism of plasma interaction with the polymer surface. Reproduced from Lu et al. (2019) [30] under conditions of the CC BY license.

Plasmas used in material processing, and in particular low-pressure plasmas used for polymer synthesis and modification, are typically maintained in a non-equilibrium state where electrons have a significantly greater energy than ions or neutrals. For the processing of polymers where bulk temperature is of critical importance, the availability of highly reactive electrons in an otherwise cold bulk gas is highly advantageous. Under these non-equilibrium conditions, exposure of the surfaces to plasmas leads to the rapid formation of a negative electric charge at the surface, facilitated by the arrival of fast electrons ahead of slow ions. This floating potential forms in the absence of any external potential, and is inherent to plasma–surface interactions. The thus-formed electric field draws ions from the plasma bulk, accelerating them toward the surface. Since ions are often the “building” or “modifying” particles for the surface, the formation of this floating potential enhances the process efficiency of surface synthesis or modification, reducing the time required for the formation of surface features, or enabling the processes that would not happen under similar conditions in the absence of plasma (Figure 1).

As already mentioned, the strength of the electric field that forms within the sheath is an effective means of control over the fluxes of energy and matter [31]. It is also important to note that when plasma comes into contact with a surface characterized by complex surface morphology (e.g., a topography with features across micro- and nano-scales), the shape of the electric field that forms between this surface and the bulk of the plasma also takes on a complex structure. In turn, the distribution of fluxes is also non-uniform across such a topographically-complex surface, with some areas receiving a greater flux of ions than others. This may be advantageous for the development of surface profiles where surface chemical and physical heterogeneity is desired, however, in other instances (e.g., the deposition of smooth uniform coatings over non-uniform surfaces), this may present a challenge. The conductivity of the substrate material also plays an important role in defining the shape and degree of non-uniformity within the thus-formed electric field, and consequently the fluxes of energy and matter from the plasma bulk to the surface [32,33,34,35]. Although outside the scope of this article, it should be noted that the formation of these electric fields and hence plasma-surface interfacial interactions become even more complex when the matter is in a liquid rather than a solid form [36,37].

Figure 1a shows some of the key processes that occur during plasma-assisted processing of the surface of a substrate. In the bulk of the plasma, the primary processes include ionization, the break down, and subsequent reassembly of molecules that leads to the formation of a variety of radicals. Once these species arrive at the substrate surface, they interact with the particles in the sheath and molecules at the surface. The outcomes of these interactions include adsorption and desorption of atoms and radicals onto the surface, abstraction of hydrogen by impinging H radical and complex radicals, direct chemically-driven adsorption of complex radicals and hydrogen atoms onto the dangling bonds, and surface transfer of the adsorbed radicals onto hydride sites as well as direct chemisorption of adsorbed radicals onto the dangling bonds. In addition to adsorption processes, the interactions with the incident ions from the plasma can lead to sputtering of material from the surface, with the process of deposition and removal of material being in constant competition with one another, where the outcome depends on the properties of the plasma and the substrate, and the experimental conditions. For polymer processing, the generation of intense UV radiation and mild heat due to ion bombardment may facilitates chain cross-linking, annealing, and desorption of loosely adsorbed low molecular weight species.

This multitude of processes provides a great deal of versatility in material processing with the same simple setup potentially being used for surface material removal (i.e., etching), addition (i.e., deposition and functionalization), and rearrangement (e.g., cross-linking). A typical diagram of plasma equipment and mechanism of plasma interaction with the polymer surface are illustrated in Figure 1b. In its simplest embodiment, this system contains a chamber with a controlled environment, often kept under vacuum for ease of ionization and to enable a greater degree of control over the reactions that take place within the plasma bulk and at the surface. A series of needle valves are used to control the injection of processing gases and monomers (in the case of material synthesis and functionalization). For systems operating under low pressure, one or more pumps are used to provide the needed level of vacuum, with high vacuum typically corresponding to a greater level of control over processes at the expense of processing time and operating cost. The pressure in the chamber is monitored using a resistance vacuum gauge. The energy for ionization is delivered into the chamber using a variety of means, with a radio frequency source of 13.56 MHz inductively coupled to the reactor being a common option (Figure 1b). In the example presented in Figure 1c, the reactor operated at the power of 18 W and pressure of about 1 Torr, with gas supply to the reactor chamber at the rate of about 8 standard cubic centimeters per minute, producing excited plasma free radicals that are sufficiently energetic to cleave bonds present in organic molecules [30]. Radicals (i.e., molecular fragments, atoms, and ions) that are generated at the result of these bond breaks can engage in subsequent reactions with molecules in the gas and on the surfaces of the solid, producing volatile species. The abstraction and addition reactions on the surface, and the resulting generation of surface radicals can be used to activate the surface to facilitate subsequent functionalization or growth of surface structures, or can be used for the break down and removal of the top layer of the material on the surface as the means of surface structuring or substrate cleaning. 

**Figure 2:**
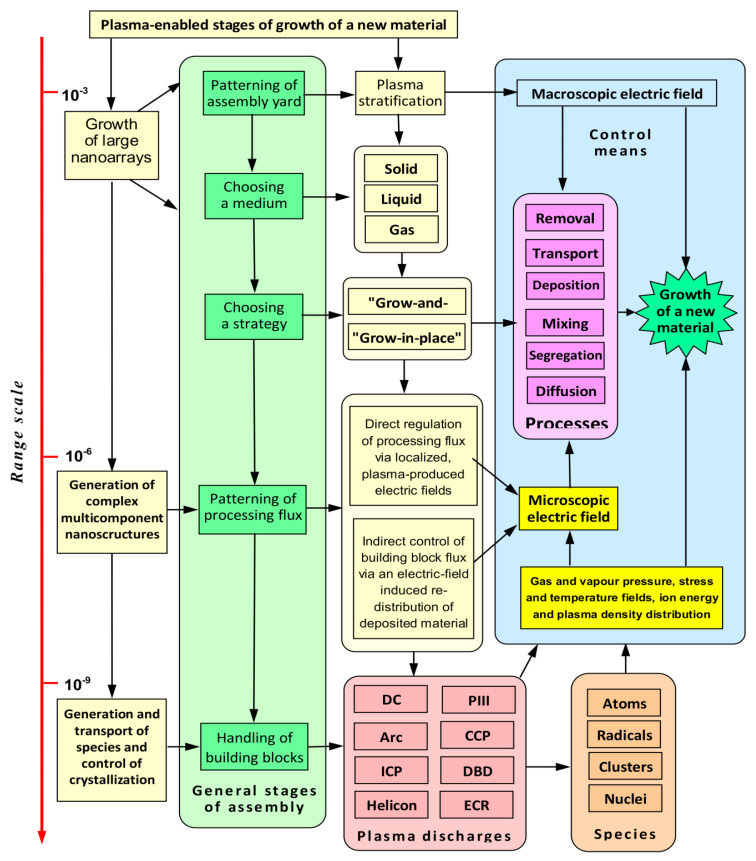
Common control mechanisms in plasma-enhanced processing of matter across multiple length scales, from particle nucleation at nanoscale, to growth of objects and arrays at micrometer and millimeter scales, respectively. Reactor geometry, energy source, and external electric and magnetic fields can be tuned to optimize the process toward specific growth and functionalization outcomes. DC refers to a direct current source, arc refers to arc plasma, ICP and CCP denote inductively and capacitively coupled plasmas, respectively, DBD denotes dielectric barrier discharges, ECR refers to electron cyclotron resonance plasma sources, and PIII refers to plasma immersion ion implantation. Reproduced with permission from Baranov et al. (2018) [34].

The treatment outcome depends on the interplay between competing processes, which can be carefully controlled through the selection of experimental design and operational parameters. Figure 2 illustrates some of the control mechanisms that can be used to modulate and tune the chemical and physical processes within the plasma environment. It should be noted that plasma is a dynamic medium, and its properties change with time. An example of this dynamic nature, which has already been explored above, is the generation of particles as a result of collisions between free electrons and positive ions with molecules in gas and on the surface, and their ongoing recombination and breakdown. Another example is the change in the structure of plasma above a surface that is being changed, particularly as the nanoscale structures form or are removed from the surface. Externally applied electric and magnetic fields are often employed to confine and structure the plasma, thus modulating the delivery of fluxes of matter and energy to enhance process efficiency by reducing losses on undesirable interactions, or enable a greater level of control over the selectivity and directionality of material removal or assembly on the surface. 

While the processing of polymers under low pressure conditions is best explored and offers the greatest degree of control, atmospheric pressure systems gain popularity because of greater treatment flexibility and ability to integrate into a wider range of industrial processes as well as ease with which these systems can be scaled up (e.g., through integration of multiple arrays of plasma jets for wide area uniform plasma treatment). When compared to vacuum systems, atmospheric pressure systems offer a lower degree of control over the surface reactions as some of the means of control available in the former have limited utility in these treatment systems. Furthermore, the energy budget required to initiate plasma is much greater than under low pressure conditions, although it should be noted that this increased energy budget is offset by the energy savings from not using vacuum pumps. As our understanding and ability to control processes that take place under atmospheric pressure conditions increases, it is likely we will see an increased uptake of these technologies across multiple sectors.

This article explores the recent progress and examples of the current state-of-the-art in the plasma-based treatment and functionalization of polymers, and outline trends that may see these techniques applied in an innovative way to improve treatment precision or deliver novel outcomes. Specifically, this article will discuss recent results, focusing on the following topics where the use of plasma-based techniques for polymer treatment have already demonstrated significant advances, namely in: (1) controlling biocompatibility and antibacterial properties of polymers; (2) hydrophilicity and hydrophobicity control of polymers; (3) enhancement of mechanical properties of polymers; (4) plasma as a means to control polymer biodegradation kinetics; (5) polymer metallization and hierarchical structuring; and (6) polymer functionalization. Finally, the article will conclude with a summary of future trends in plasma technology for the modification and functionalization of polymers.

## 2. Plasma for Enhancing Biocompatibility and Antibacterial Properties of Polymers

**Figure 3:**
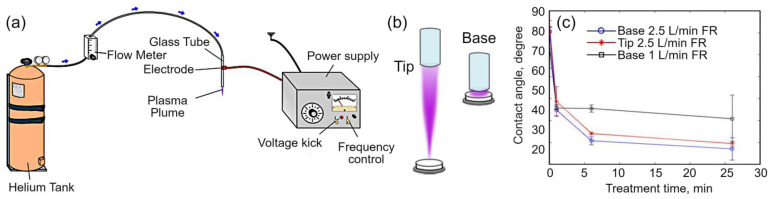
(**a**) Schematic of cold atmospheric plasma (CAP) source used for the treatment of the ultra-high-molecular-weight polyethylene (UHMWPE) samples. (**b**) Tip and base treatment locations of the UHMWPE samples in the plasma plume. (**c**) Contact angle measurements as a function of the treatment time varied from 1 to 26 min. Reproduced from Turicek et al. (2021) [38] under conditions of the CC BY license.

The use of plasma to control protein-surface and cell-surface interactions of biomedically-relevant polymers has been well explored, both at low pressure and in atmospheric pressure plasma systems. For example, biocompatibility enhancement has been achieved by treatment of ultra-high molecular weight polyethylene (UHMWPE) by cold atmospheric plasma (CAP) [38]. Frequently used in artificial joints, UHMWPE has an attractive combination of chemical inertness under biological conditions as well as low friction that facilitates the use of this polymer in reticulating applications [37]. At the same, this polymer is susceptible to mechanical wear, especially in the presence of wear debris, and has suboptimal adhesion and lubrication characteristics. To address these challenges, Turicek et al. [38] used a tubular helium CAP source to tune wear resistance, wettability, and surface adhesion properties of the UHMWPE material. The team of authors demonstrated that the properties could be tuned by varying the distance between the treated surface and the nozzle (hence exposing the surface to different sections of the plasma plume) as well as by adjusting gas flow rates and duration of exposure. A single treatment was able to reduce the water contact angle by 32–54° for the plasma treatment times up to 26 min, and enhance the root mean square surface roughness by up to ten fold, corresponding to changes in surface lubrication and adhesion properties. Importantly, it was possible to attain these significant changes in the surface properties without affecting durometer hardness of the material. The scheme of the experiment as well as plasma plume regimes and some results are shown in Figure 3a–c. While the surface lubrication and adhesion were not directly tested and measured in this study, the obtained wettability and hydrophilic properties assume the increased lubrication and adhesion, as stated in the original publication [38] and in the several references cited there.

**Figure 4:**
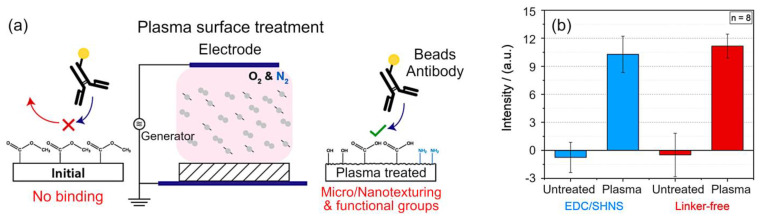
(**a**) Exposure of polymer surfaces to plasma species leads to surface texturing, with features spanning micro and nano-scales, and simultaneous introduction of functional groups with an affinity for biomolecules, where the latter could be selectively immobilized via physical and covalent bonding in the absence of the linker. (**b**) Antibody immobilization on pristine and plasma-treated poly(methyl methacrylate) surfaces: (i) 1-Ethyl-3-(3-(dimethylamino)propyl)carbodiimide/N-hydroxysulfosuccinimide (EDC/sHNS) mediated and (ii) linker-free immobilization of antibodies labelled using Au nanoparticles. The mouse IgG antibodies labelled with two different methods were used. Reproduced from Wieland et al. (2020) [40] under conditions of the CC BY license.

The surface properties of poly(methyl methacrylate) have also been significantly modified by Wieland et al., who demonstrated an enhancement in protein immobilization on plasma treated polymers as a result of plasma surface activation [38]. Through process optimization, a treatment protocol that uses a gas mixture of half-and-half of oxygen and nitrogen as the processing gas, the input power of 1000 W, and the treatment duration of 5 min was found to produce the highest increase in immobilization efficiency coupled with the most uniform distribution of attached biomolecules across the polymer surface. The substrate temperature under these conditions was measured to reach 80 °C. The scheme of the experiment is illustrated in Figure 4a. It is a well-documented observation that under ambient conditions, PMMA polymers tend to show a low surface energy, attributed to the presence of non-polar hydrogen bonds on their surface. When exposed to oxygen plasma-generated species including UV radiation, polymer chains undergo a break down and simultaneous functionalization (with oxygen species derived from the plasma as well as from the ambient air), resulting in the surface enriched by oxygen radicals. This, in turn, leads to increased wettability of the treated surfaces. Addition of nitrogen species into the gas mixture or by using pure nitrogen plasma can facilitate the introduction of such reactive groups as amines (NH_2_) and carboxyl groups (–COOH). Once functionalized, the surfaces can be exposed to relevant biomolecules to promote their immobilization; this process can be done either in the presence or in the absence of a linker (e.g., 1-Ethyl-3-(3-(dimethylamino)propyl)carbodiimide/N-hydroxysulfosuccinimide (EDC/sHNS)). The latter are frequently used as mediators for bioconjugation of carboxylated peptides and small proteins [39]. The comparison of immobilization techniques that do or do not involve the EDC/sHNS linker is illustrated in Figure 4b. The mouse IgG antibodies labelled with two different methods were used.

**Figure 5:**
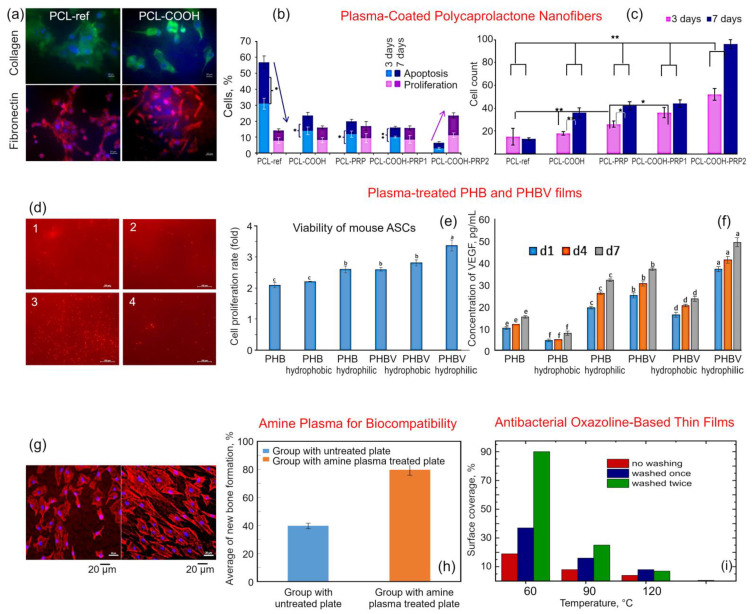
(**a**) Fibronectin (FN) and collagen secretion by the fibroblasts seeded on PCL-ref and PCL-COOH. Cells were stained by antibody Alexa Fluor 594 (orange) to collagen (magnification 40×) and Alexa Fluor 488 to FN (magnification 20×). (**b**,**c**) The influence of scaffold surface on (**a**) cell proliferation and apoptosis, and (**b**) cell count for three and seven days of cultivation. The arrows indicate the relationship of the level of cell apoptosis and cell proliferation. Reproduced from Miroshnichenko et al. (2019) [41] under conditions of the CC BY license. (**d**) Cell adhesion of in vitro cultured mouse ASCs onto various plasma-treated films. (**e**) Cell proliferation rate of in vitro cultured mouse ASCs on PHB, PHB hydrophobic, PHB hydrophilic, PHBV, PHBV hydrophobic, and PHBV hydrophilic surfaces. (**f**) Concentrations of VEGF secreted from in vitro cultured mouse ASCs on PHB and PHBV films over a 7-day period. Reproduced from Chang et al. (2018) [42] under conditions of the CC BY license. (**g**) Morphologies of cells on the surfaces of plates: untreated titanium plate (left) and titanium plate treated with amine plasma (right). Nuclei color is blue. (**h**) Comparison of the average percentage of new bone formation for each specimen in the animal experiments. Reproduced from Jeong et al. (2019) [43] under conditions of the CC BY license. (**i**) Bacteria *S. epidermidis* surface coverage area (percent) formed on POx films deposited at different substrate temperatures. Reproduced from Stahel et al. (2019). [44] under conditions of the CC BY license.

Miroshnichenko et al. studied the plasma-enhanced adhesion and growth of human fibroblasts on plasma-coated polycaprolactone (PCL) nanofibers [41]. Figure 5a illustrates the fibronectin (FN) and collagen secretion by the fibroblasts seeded on PCL-ref and COOH plasma-modified polymers. Figure 5b,c illustrate the influence of scaffold surface on cell proliferation and apoptosis, and cell count for various times of cultivation. Apparent differences between the groups are visible, and the enhancement is significant. As seen in Figure 5b,c, the rates of apoptosis and proliferation were significantly (by about 50%) affected by plasma treatment for both three and seven days incubation periods. However, the experimental system used to achieve this enhancement in the attachment of cells to PCL nanofibers is uncomplicated, and involves a two-step protocol of COOH functionalization of surfaces followed by exposure to the EDC cross-linker and covalent attachment of proteins via NH_2_ groups. Despite its simplicity, the process allowed for efficient immobilization of active molecules from the platelet rich plasma (PRP) while maintaining their biochemical activity, which allowed for the subsequent increased fibroblast attachment, survival, and proliferation on PCL-COOH-PRP2 samples when compared to the control samples (PCL-ref), or samples that were only subjected to plasma treatment (PCL-COOH) where no cross-linker was used. These results suggest that this simple plasma-enabled enrichment of surfaces with COOH functional moieties can expand the use of PCL-based biomaterials in tissue engineering.

The effect of plasma treatment on the biocompatibility of polymers produced by means of fermentation of glycerol by *Ralstonia eutropha H16*, a Gram-negative lithoautotrophic bacterial species from the β-subclass of the Proteobacteria, was studied by Cheng et al. These studies revealed an increase in the number of chemically-active moieties, and a change in the wettability of treated PHB and PHBV films, with the nature of contact angle change dependent on the chemistry of the processing gas used for the treatment. Specifically, CH_4_/O_2_ treatment of poly(3-hydroxybutyrate) (PHB) and poly(3-hydroxybutyrate-co-3-hydroxyvalerate) (PHBV) resulted in a significant decrease in water contact angle from 76.0° and 72.5° to 23.0° and 18.5°, respectively, whereas C_2_H_2_F_4_ plasma treatment of the same films increased the contact angle to 134.0° and 158.5°, respectively. When the thus-modified surfaces were investigated with respect to their ability to support the attachment and growth of mouse adipose-derived stem cells (ASCs) under in vitro conditions, PHBV films treated with CH_4_/O_2_ demonstrated the best performance, followed by PHBV films treated by C_2_H_2_F_4_ and unmodified PHBV films. While plasma treatment improved cell attachment and viability on PHB surfaces, the results were inferior to those attained for PHBV films. The cell adhesion of in vitro cultured mouse ASCs on various plasma-treated films is shown in Figure 5d, the cell proliferation rate of in vitro cultured mouse ASCs is shown in Figure 5e, and concentrations of VEGF secreted from in vitro cultured mouse ASCs on PHB and PHBV films is presented in Figure 5f.

Thus, these studies provide evidence that plasma-based polymer treatment is a useful technology for biomaterial development and expanding applications of promising hydrophobic polymers into medical fields, particularly for enhancing the biocompatibility and fine-tuning wettability of inherently hydrophobic PHBV, which has lower crystallinity and greater flexibility when compared to more rigid PHB, and hence is more amenable to processing and application in the biomedical field. The wide range of surface chemistries, topographies, and contact angles that can be easily attained via plasma modification on the same polymer material is a highly valuable feature of plasma treatment, since it allows for optimizing the wettability and surface chemistry to stimulate protein and cell attachment for any given cell type.

In addition to using plasmas to modify the properties of bulk polymers and macroscopic polymer films, plasmas can be used to deposit thin layers of bioactive polymers onto the surfaces of substrates, from metals to ceramics and composites. Jeong et al. used amine plasma treatment to improve the biocompatibility of titanium maxillofacial plates (Figure 5g,h). Deposition of thin plasma polymer films rich in amine functional groups has been demonstrated to be an efficient technology to introduce nitrogen-containing moieties onto the surfaces of metals. Thus-treated Ti implants demonstrated improved hydrophilicity and biocompatibility under in vitro and in vivo conditions using MC3T3-E1 osteoblast cells and rabbits as respective model organisms. Improved osteoblast differentiation and calcification was reported on plasma-treated Ti surfaces with histological findings from in vitro studies showing greater uniformity and length of new bone formed in contact with plasma-treated Ti surfaces [43].

Several polymers deposited under plasma conditions have demonstrated an attractive combination of antibacterial properties and cytocompatibility performance. For example, our group has been investigating the use of essential oils and their components for the fabrication of such coatings [3] to harness the broad spectrum of activity and variable target mechanisms of these antimicrobial agents [45]. Polymer coatings fabricated from terpinene-4-ol and its parent tea tree oil [46,47], geranium [48], and other plant secondary metabolites [49] under low pressure conditions in weakly ionized radiofrequency plasmas were effective in reducing the attachment, proliferation, and biofilm formation by Gram-positive and Gram-negative bacteria while allowing for improved attachment of mammalian cells including osteoblasts, fibroblasts, monocytes, and others. Their efficacy could be further enhanced by introducing other antimicrobial agents in their structure (e.g., in the form of ZnO particles entrapped within the plasma polymer film) [50]. Importantly, these composites were produced using the very same system used for material deposition. An increase in the biochemical activity of coatings was also observed when the pulsed plasma regime was used [51,52] as it facilitated the retention of desirable chemistries within the film, in a similar manner that reducing input power prevents excessive monomer fragmentation [53]. Unlike many of the other plasma polymers, essential-oil derived plasma polymers fabricated by our group were optically transparent and stable when treated using UV light and gamma irradiation at doses typically used in medical settings for sterilization [54,55,56,57].

Polymer films polymerized under atmospheric pressure conditions have also been reported. For example, Stahel et al. demonstrated oxazoline-based thin films fabricated from 2-methyl-2-oxazoline vapors under nitrogen plasma conditions preserved the highly desirable oxazoline rings in the structure of resulting polymer [44], with these rings typically lost under conventional oxazoline polymerization. The results of these studies are shown in Figure 5i. The chemistry of the resulting films, and consequently their ability to support the attachment and viability of mouse embryonic fibroblast cells while retarding fouling by *Staphyloccocus epidermidis*, *Staphylococcus aureus*, and *Escherichia coli* was found to depend on the substrate temperature, with films deposited at T_substrate_ = 120–150 °C showing superior performance.

**Figure 6:**
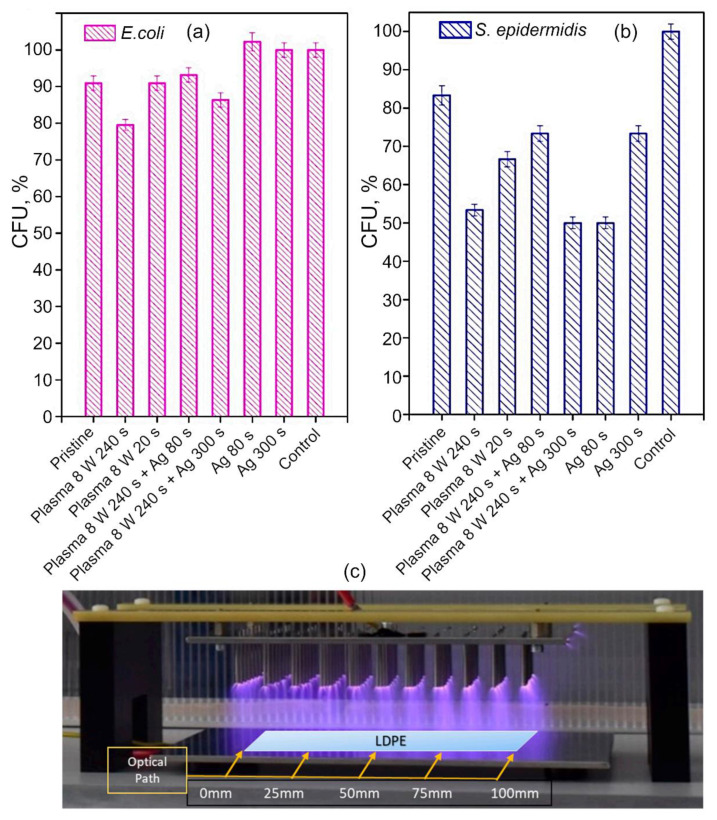
(**a**,**b**) Combination treatment that involves plasma treatment followed by Ag sputtering improved antimicrobial activity of polytetrafluoroethylene (PTFE) nanotextiles against Gram-negative *E. coli* and Gram-positive *S. epidermidis*. Plasma treatment was performed at the input power of 3–8 W for up to 240 s, and the Ag sputtering was performed for 80–300 s. The samples were allowed to incubate in the presence of bacterial culture for 2 h, and then cells were left to grow overnight on agar plates. Reproduced from Slepicka et al. (2021) [58] under the conditions of the CC BY license. (**c**) The non-thermal plasma pin system used to deliver plasma treatment uniformly over larger surface areas. Not seen on the image is a plastic box that was used to cover the system during treatments and optical measurements. The low-density polyethylene (LDPE) samples were placed within the plasma discharge for the duration of the treatment. Shown is the plasma discharge in ambient air. Reproduced from Scally et al. (2018) [60] under conditions of the CC BY license.

Figure 6 illustrates the antimicrobial activity of plasma-activated polytetrafluoroethylene (PTFE) nanotextile samples subsequently decorated with Ag against bacterial strains of *E. coli* (Figure 6a) and *S. epidermidis* (Figure 6b) [58]. From the results, it is evident that the combination of plasma treatment and sputtering of silver is an effective means to enhance the surface’s ability to resist colonization by *S. epidermidis*. Interestingly, the effect of this combination treatment on settlement by *E. coli* was far less pronounced, with the surface receiving 8 W plasma treatment only for 240 s, showing the greatest reduction in viable colony forming units, followed by a surface subjected to the same plasma treatment followed by Ag sputtering for 300 s. This example highlights two challenges in the development of plasma coatings. One concerns the difficulty in predicting the optimum conditions under which the greatest level of antimicrobial performance can be attained. The second one concerns the difficulty of designing a surface that would effectively inhibit attachment and biofilm formation of different species of microorganisms. It is not uncommon for Gram-positive and Gram-negative cells, or cells of different sizes, shapes, and charges to react differently to the same surface.

Biodegradation of the polymers treated using plasma is another important aspect of the biocompatibility of medical polymers and coatings, where the treatment can be applied to intentionally render the substrate material more susceptible to biodegradation via surface activation to accelerate its break down and dissolution, or in contrast, slow down the degradation in materials known to break down prematurely under in vivo conditions. An example of the latter is the use of thin polymer films to slow down the degradation of magnesium bioresorbable implants, since in their uncoated form, these materials undergo premature loss of mechanical strength due to in vivo corrosion [59]. Outside the medical field, plasma treatment can render waste polymers more amenable to biodegradation under ambient conditions, with the aim to reduce ecosystem pollution associated with the use of commercial plastics. For example, Scally et al. used plasma to induce changes in the surface chemistry of low-density polyethylene (LDPE) to facilitate their subsequent break down using *P. aeruginosa*. In this study, low-temperature plasmas generated in ambient air with an admixture of CO_2_ at the ratio of 25:1 was used for the treatment (Figure 6c). Optimization of the treatment process identified the voltage of 32 kV and treatment time of 300 s to be most conducive to inducing the necessary changes in the properties of the polymer, reflected by the greatest increase in polymer break down after this treatment [60].

From these examples, it is evident that deposition and modification of plasma polymers on the surface of materials has a significant potential across many fields where interactions with biomolecules or cells is anticipated, from medicine, to the control of marine fouling [61,62,63,64], manage the reactivity of coatings [65,66,67], and waste management. The examples of plasma use in these fields are numerous, and include enhancement of the biocompatibility of cyclopropylamine-based polymers on meshes constructed of poly (β-caprolactone) nanofibers [68], for the synthesis of the nitric oxide releasing polymeric coatings [69], and other biomedical and antibacterial applications [70,71]. In addition to searching for new polymers and applications, further work is currently in progress to design more efficient and selective plasma-based methods for material processing [72].

What becomes clear from examining the aforementioned case studies is the wide range of materials and outcomes that can be attained within the existing range of plasma tools, which continues to expand to include ever more sophisticated processing environments. The combination of surface chemistry, topography, and stiffness define the in vitro and in vivo performance of materials, from protein adsorption and mammalian cell growth to bacterial colonization and biodegradation. In addition to controlling the properties that define *nonspecific* protein- and cell-material interactions at the surface, plasma treatment can serve as a functionalization step for the development of surfaces with bound biological epitopes where the attachment is highly specific. Importantly, certain plasma-based treatments may enable the modification of surface topography and compliance that is decoupled from surface chemistry, providing an additional degree of control for cell-surface systems where separating these modifications is critical.

## 3. Plasma for Polymer Functionalization and Etching

**Figure 7:**
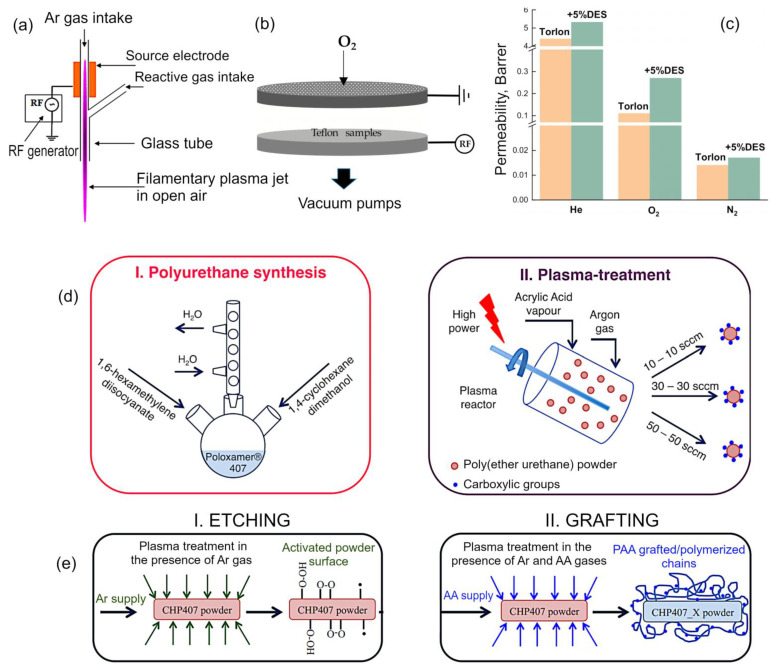
(**a**) Configuration of the dielectric barrier discharge Y-shaped source. Reproduced from Panaitescu et al. (2020) [73] under conditions of the CC BY license. (**b**) Scheme of the electrode system of the plasma reactor setup used for plasma nano/microtexturing of Teflon samples. The upper electrode is a shower-head type to allow homogeneous feed of the process gas (O_2_ in this case). Samples are placed on the bottom electrode, which is radio-frequency (RF) powered. Reproduced from Mundo et al. (2017) [74] under conditions of the CC BY license. (**c**) Permeability of Torlon and Torlon/DES-5 composites for He, N_2_, and O_2_ at 30 °C. Reproduced from Pulyalina et al. (2021) [75] under conditions of the CC BY license. (**d**) Schematic representation of the two main steps needed for the design of an amphiphilic poly(ether urethane) exposing a tunable amount of carboxylic groups: (I) polyurethane synthesis; (II) powder plasma treatment. (**e**) Schematic representation of the plasma treatment process: (I) etching phase in the presence of Ar gas to create free radicals on powder surface and (II) grafting step in the presence of Ar gas and acrylic acid vapors to expose carboxylic groups. Reproduced from Laurano et al. (2019) [80] under conditions of the CC BY license.

Another field where plasma-based methods play a prominent role is the functionalization of polymers. Panaitescu et al. demonstrated that use of plasma-based techniques can provide superior functionalization outcomes when compared to conventional chemical methods when modifying microcrystalline cellulose (MCC). For plasma treatment, the team chose to use liquid, delivering oxygen flow by means of a Y-shaped tube (Figure 7a). For conventional treatment, a 2,2,6,6-tetramethylpiperidine-1-oxyl radical (TEMPO) mediated oxidation was used. While in both cases, cellulose was successfully functionalized, TEMPO oxidation resulted in the loss of thermal stability in MCC, which was shown to reduce by 80 °C. In contrast, plasma treatment delivered a gentler oxidizing effect, corresponding to better thermal stability of thus-treated MCC. The composite made using treated cellulose and poly(3-hydroxybutyrate) had a significantly altered morphology and physical properties when compared to the composite made using unmodified MCC, with a pronounced improvement in the strength of the interface between MCC fibers and the polymer matrix, and as a result, better mechanical properties in the samples employing plasma treated fibers [73].

Generally speaking, surface functionalization is typically performed to change the nature of the interactions that take place at the interface when the said surface comes into contact with matter, be it a living tissue, solid, liquid, or gas. In the previous section, a number of examples where surface functionalization was used to define protein–surface and cell–surface interactions, and surface–fluid interactions were discussed with the view of their applications in medical settings. However, the interactions between surfaces and liquids are critical in many other fields, from designing self-cleaning and low-drag surfaces, to ensuring the deposition of heterogeneous thin films during spin coating to name but a few. The chemistry of the surface, along with its surface roughness, define the wetting and adhesion properties of the surface, thus surface functionalization is commonly used to render surfaces more or less wettable. For example, Mundo et al. used plasma modification to make the surface of Teflon more repellent to water droplets in air, and to reduce the drag when the material is used in underwater applications [74]. Teflon is an inherently hydrophobic material. In this example, oxygen plasma etching for 10 min under vacuum was used to impart a complex surface architecture onto Teflon surfaces that was similar to that of the surface of the leaf of *Salvinia molesta*, an aquatic free-floating fern. The experimental setup, although simple, allowed for the processing of sheet and spherical Teflon substrates. Scheme of the electrode system of the plasma reactor used for the plasma nano/microtexturing of the Teflon samples is illustrated in Figure 7b. The exposure to oxygen plasma resulted in the formation of tall and thin cusps with the average size of ~5.5 µm spaced at ~1.5 µm, which were in turn decorated with even finer filaments with the diameter to the order of tens of a nanometer. Because the plasma etching process used in this experiment can be described as stochastic, there is an evident randomness in the dimensions, shape, and distribution of thus-developed surface structures across the surface of the Teflon substrate. It should be noted that the stochasticity arises from the mechanism by which masking is produced in this system. When plasma is initiated, the bombardment of energetic ions against the surface of electrodes leads to sputtering of particles of metal and their subsequent random deposition on the surface of Teflon. The polymer material not masked by the metallic particles is preferentially etched away in the oxygen plasmas, producing a random pattern of surface features that have a tendency to trap air. This layer of trapped air is retained when the material is immersed in water under both static and dynamic conditions (e.g., when the Teflon sphere is dropped vertically in the pool of water), or when droplets of water are dispersed onto the surface in air. In the latter case, the intensity with which the incident water droplets are repelled by the surface is comparable to that observed on heated solid objects.

Where the previous example demonstrated the use of plasma treatment to change the nature of liquid–solid surface interactions, the very similar principle can be used to define how surfaces interact with gases, with an immediate application in gas separation. There are a number of polymers that show excellent selectivity in separating gas mixtures. For example, membranes fabricated from polyamide–imide Torlon and functionalized with deep eutectic solvent (1:3 M of zinc chloride to acetamide) can effectively separate the O_2_/N_2_, H_2_/N_2_ and He/O_2_ mixtures (Figure 7c) [75]. Plasma treatment can be used to modify the topmost layer of the membrane (via functionalization or deposition of an ultra-thin layer) to render it highly selective, while leaving the strong porous substrate virtually unchanged [76,77,78]. In a very similar manner, membranes used for energy applications, water purification, and separation of liquid mixtures can be modified and their performance enhanced using plasma treatment [79].

The examples thus far have primarily discussed the use of plasma to modify the properties of materials that have already been shaped into a ready-for-use product (e.g., as a scaffold, a membrane, or a component of medical implant). However, plasma processing of precursors to these final products during earlier steps of production offers certain advantages. For example, exposure of powders to plasma treatment may significantly increase the availability of chemical moieties on the surfaces of particles, in turn increasing the overall availability of reactive functional groups within the material (when compared to the unmodified material or where plasma treatment was only applied to the surface of the ready-made polymer). While plasma treatment of powders is not trivial if one wants to attain modification uniformity, and balance the level of functionalization against particle clumping, these powders can find a wide range of applications from being used in inks and paints to active ingredients in cosmetics and as building blocks for biomedical hydrogels and thermoplastics.

Laurano et al. studied the possibility of functionalization of amphiphilic polyurethane by plasma treatment of polymer powder [80]. In this study, powders of poly(ether urethane) containing Poloxamer^®^ 407 blocks (M_W_ = 54,000 Da) were subjected to argon plasma treatment to introduce active sites onto the surface of the particles, and then exposed to the vapors of acrylic acid to functionalize these active sites with a -COOH group. The result of this treatment was the introduction of carboxylic groups along poly(ether urethane) polymer chains. This was done to demonstrate the possibility of imparting stimuli responsiveness, specifically basic pH sensitivity in this case, in the hydrogels produced using these powders. These carboxylic groups can also serve as sites for controlled cross-linking, and for covalent attachment of biomolecules to control hydrogel interactions with cells. The degree of functionalization and chain stability upon plasma exposure varied with the argon gas flow, with greater flow rates associated with a notable chain degradation and loss of material (with a loss of 35% molecular weight for the flow rate of 50 sccm). The presence of Poloxamer^®^ 407 blocks allowed the aqueous solutions containing the plasma treated polymer to retain its levels of thermoresponsiveness. This process can be extended to other polymers and functional groups, providing a means to expand the multifunctionality of hydrogels. Figure 7d shows the schematic representation of the two main steps needed for the fabrication of amphiphilic poly(ether urethanes), and Figure 7e shows the schematic representation of the plasma treatment process used to introduce carboxylic groups along the polymer chain.

Plasma-based technology appears to be an efficient environment for the functionalization of various polymers, as follows from the above outlined examples and other applications such as activation of nanowires for electrocatalytic sensing of nitrate in food [81], activation of polylactic acid-based wood-plastic composites [82], improved polypyrrole adhesion [83], and many others. The wide diversity of experimental setups that can be enhanced using plasma sources provide a great deal of flexibility, while the use of variable gas chemistries and their mixtures allow for surface functionalization of virtually any type of flat or 3D object with an equally broad range of functional groups. Placement of the treated object can be used as an additional mechanism of control over the rate and chemistry of the reaction, and combination with pre- and post-treatment provide an additional avenue for attaining complex chemistries. Unusual surface chemistries can also be attained using plasma treatment, as high reactivity of the environment can enable and promote reactions that would not take place under similar conditions in the absence of plasma. Importantly, as a treatment that takes place at low (e.g., room) temperature and in the absence of liquids or harsh chemicals, plasma treatment can be used on implants and devices in their final stage of preparation (e.g., fully shaped and/or assembled) without any damage to the bulk material or fine elements that comprise a device.

## 4. Plasma for Hydrophilicity and Hydrophobicity Control of Polymers

**Figure 8:**
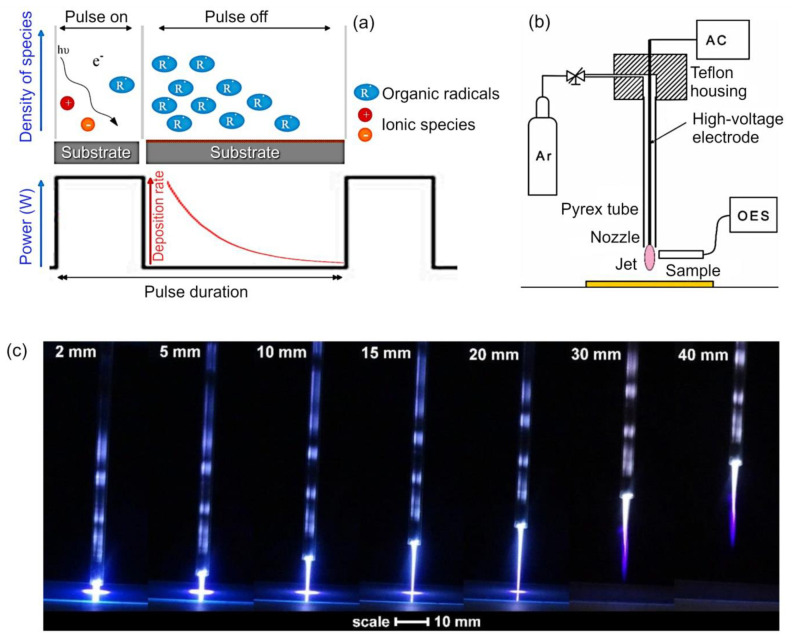
(**a**) Schematic representation of polymer deposition during pulse-on and pulse-off pulsed mode in plasma polymerization. Reproduced from Iqbal et al. (2018) [84] under conditions of the CC BY license. (**b**) Schematic drawing of the atmospheric pressure plasma jet system used for polymer surface modification, and (**c**) optical photograph of the plasma jet at various distances of the nozzle from the sample. Reproduced from Vesel et al. (2020) [87] under conditions of the CC BY license.

In previous examples, we have already briefly discussed plasma technology as a tool that can deliver efficient control of hydrophilicity and hydrophobicity of polymer surfaces. It should be noted that for many of these examples, it is necessary to use plasmas that are sufficiently reactive to induce the desired degree of functionalization or structuring of the surface, yet are gentle to enable selectivity and preserve the chemistry of the functional groups that are being attached onto the surface. This can be achieved using plasmas generated at very low input powers, however, often under these conditions, it is difficult to produce stable coatings. Another approach centers on the use of pulsed rather than continuous wave modes of treatment, because these plasma conditions produce a favorable combination of efficient surface activation and grafting of building/functionalization blocks, while preventing excessive monomer fragmentation and their random recombination. The polymers produced under pulsed mode conditions often display a more regular structure and a greater degree of original monomer chemistry when compared to polymers produced under continuous mode at very low input power. Figure 8a illustrates the polymer deposition during pulse-on and pulse-off pulsed mode. During the former mode, the surface is activated through ion bombardment and UV irradiation, and the molecules of the monomer in the gas phase are broken down into fragments, some of which recombine, with the degree of fragmentation and recombination dependent on experimental parameters such as gas flow, chamber pressure, and input power. The “plasma on” mode typically lasts from microseconds to milliseconds, and is followed by an “plasma off” mode, which is designed to reduce the overall power density of the process, and facilitate more controlled reactions between free radicals generated during the “plasma on” mode and the formation of a more regular polymer structure [84].

It should be noted that where in most examples in the literature, pulsed plasma polymerization is performed in low pressure reactors, there are examples of pulsed plasma polymerization under atmospheric pressure. For example, Wang et al. used nanosecond impulse discharges to drive the polymerization of pyrrole on the surface of the pyrrole microemulsion [85]. The properties of the resultant polypyrrole self-supporting thin films (e.g., their thickness, conductivity, and roughness) were determined by operating parameters such as discharge length and intensity, flow of processing argon gas, and the presence of nitrogen gas. The mechanism of polymer formation under these conditions is significantly different from that on the solid surface from volatile precursor, and to that during atmospheric aerosol assisted pulsed plasma polymerization [86]. Briefly, the collisions between plasma-generated electrons and N_2_ molecules in air results in the generation of nitrogen-containing species including free radicals, some of which reach the interfacial layer between the gas and liquid phases, diffusing across this layer into the microemulsion. There, they are able to oxidize pyrrole molecules, generating pyrrole cation radicals that can polymerize first into polymer clusters with a rod shape, and with time into cross-linked polymer sheets. It is important that this cross-linking stage is given sufficient time and there is an ongoing supply of nitrogen radicals, since reduction in either of these parameters leads to films of suboptimal quality, with rod-shaped particles loosely embedded onto the surface of the film rather than properly integrated into the film polymer matrix. The chemical composition and surface morphology of these films was affected by the selection of Ar gas flow and the number of plasma discharge used for polymer fabrication, where the number <10,000 resulted in smoother polymers whereas number of discharge times >10,000 produced porous polymers.

Another important aspect of plasma treatment under atmospheric pressure conditions is treatment uniformity and unwanted chemical modifications and thermal treatment that may arise during wettability treatment. Vesel et al. investigated the suitability of an atmospheric-pressure plasma jet produced using a high-impedance low-frequency discharge in wet argon as a means of imparting desired wettability onto polyethylene terephthalate (PET) with minimal detrimental effects [87]. The authors demonstrated that the distance between the nozzle of the jet and the surface of the sample as well as treatment duration played a defining role in balancing desirable changes in wettability against undesirable effects. Keeping the distance small allowed the researchers to attain the desired level of wettability rapidly and over areas of several cm^2^. In contrast, increasing the distance between the nozzle and the surface even to several mm led to a substantial reduction in the level of functionalization for the same time, requiring substantially longer processing times to achieve comparable levels of functionalization. Importantly, closer examination of surface wettability under these different treatment regimens showed a great degree of spatial and temporal non-uniformity. The area corresponding to the center of the plasma plume showed the greatest reduction in the contact angle, with the reduction becoming less pronounced as one moves toward the edges of the treated area. With treatment time, the area of increased wettability undergoes an outward growth, yet for the outer regions of the treatment area, it never quite reaches the desired levels of wettability, even after extended treatment. For example, one sample showed a central area with a contact angle of 25°, surrounded by a transition band of approximately 5 mm in width, where the contact angle changed monotonically from 25° to 70° in an outward direction. These bands of highly wettable and virtually unmodified areas are a result of gradients of reactive species that exist in both axial and radial directions, and present both a challenge with respect to attaining a uniform modification, but also an opportunity for the fabrication of more complex surfaces where heterogeneity and sharp transition between zones of high and low wettability are desirable. Figure 8b illustrates the schematic drawing of the atmospheric pressure plasma jet system used for polymer surface modification, and Figure 8c is the optical photograph of the plasma jet at various distances of the nozzle from the sample.

Plasma-based technology is quite efficient for the active control of polymer hydrophilicity and hydrophobicity, as evidenced by the above outlined examples. Moreover, extensive research efforts are currently underway to improve the efficiency and controllability of polymer wettability characteristics [88,89]. In addition to how surfaces interact with water, plasma treatment can be used to adjust oleophobicity. Polymers that are both hydrophobic and oleophobic are important. At one end of the possible spectrum of applications for such polymers are reusable food packaging such as bottles, containers, and bags, where the retention of liquid food residue may be undesirable. Clearly, for these applications, treatment setups that can sustain scaled up treatment are desired, and minor variations in treatment uniformity may be acceptable. At the other end of the spectrum of applications are microchannel devices (e.g., highly sensitive microfluidic wearable sensor for detection of molecules or intraocular pressure monitoring), where modification needs to be applied within a highly confined environment but with much greater level of precision.

## 5. Plasma for Modifying Mechanical Properties of Polymer Composites

**Figure 9:**
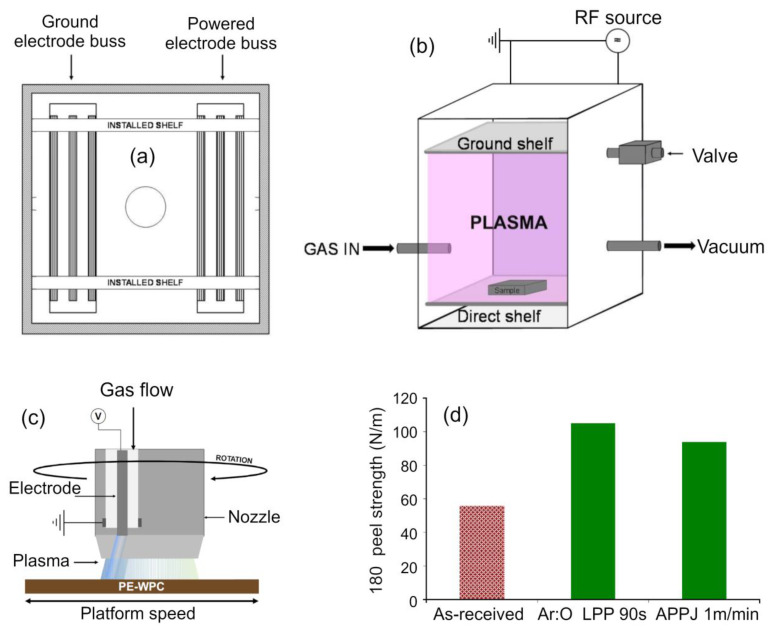
(**a**,**b**) Schematic of a low-pressure plasma (LPP) reactor used for the surface treatment of polyethylene wood plastic composite (PE-WPC) and chamber scheme and (**b**) the direct configuration scheme. (**c**) Schematic of an atmospheric pressure plasma jet (APPJ) system used for the surface treatment of PE-WPC. (**d**) The 180° peel strength values for an as-received and plasma-treated PE-WPC/acrylic adhesive joints. An adhesion failure always occurred. Reproduced from Yáñez-Pacios et al. (2018) [90] under conditions of the CC BY license.

Modification of the mechanical properties of polymers is one more application of plasma-based techniques where the use of plasma enables high efficiency and yield. Figure 9 illustrates the technology and results obtained by Yáñez-Pacios et al. when modifying the properties of wood plastic composites (WPCs), materials that result from adding particles of ground wood into a thermoplastic resin [90]. In general, these composites offer a number of advantages over wood such as improved resistance to decay and rot, moldability into desired shapes, and provide an opportunity to use this natural resource more efficiently. At the same time, these composites have lower mechanical strength and stiffness when compared to wood as well as limited surface adhesion, which prevents the use of adhesives to join components made of WPCs and of paints to change the visual appearance of the material once it has been installed. The low surface adhesion is the result of the use of non-polar polymers (e.g., poly (vinyl chloride)). In order to facilitate the use of adhesives and thus enable lamination of polyethylene-based WPC components, Yáñez-Pacios et al. exposed polyethylene-based WPC surfaces to plasmas generated under low (Ar:O_2_ at 2:1, LPP) and atmospheric pressure (synthetic air APPJ). Both types of treatment resulted in a substantial improvement in the adhesion properties of WPC as tested using acrylic adhesive tape, attributed to the surface modification through chemical functionalization and ablative structuring. The changes in the surface properties were found to be transient, as commonly reported for plasma treated polymers due to the ability of polymer chains to reorient themselves to minimize surface free energy when in contact with ambient air. Thus, for surfaces allowed to age in ambient air for >24 h prior to application of the adhesive, the adhesion strength was lower when compared to surfaces where the adhesive tape was applied immediately after plasma treatment. In the latter case, there was no hydrophobic recovery and the strength of adhesion remained stable and in some instances showed an increase over time. Given that the improvements in the adhesion performance was similar for atmospheric pressure and low pressure plasma systems, there is a pathway to translate these findings into real-life applications.

**Figure 10:**
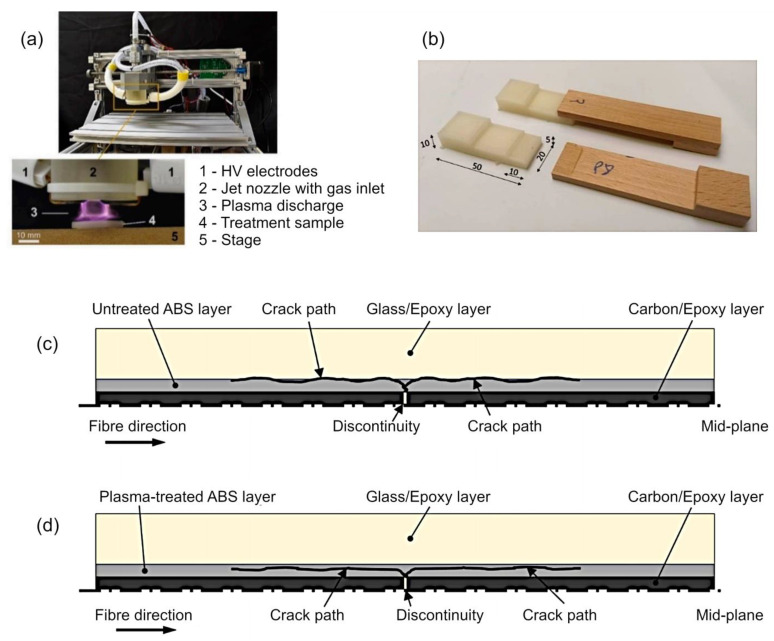
(**a**,**b**) Plasma device used for the surface treatment of 3D-printed samples on a CNC positioning system (top left image) with a gliding arc plasma jet (**a**), and sample for testing the bond shear strength (**b**). Reproduced from Kariž et al. (2018) [91] under conditions of the CC BY license. (**c**,**d**) Representation of the crack path in ABS-interleaved hybrid composites (**c**) with untreated ABS (**d**) with plasma-treated ABS. Reproduced from Marino et al. (2020) [92] under conditions of the CC BY license.

Figure 10a,b illustrates a plasma device used for the surface treatment of 3D-printed samples on a computerized numerical control (CNC) positioning system with a gliding arc plasma jet, and a sample for the testing of the bond shear strength developed by Kariž et al. [91]. 3D printing, or additive manufacturing, enables the production of objects and structures with precise geometric shapes in prototyping and, increasingly, in the industrial scale production of goods. However, most of the materials typically used in 3D printing have not been designed to bond readily with adhesives used in woodworking. This often necessitates an extra step of surface preparation aimed to enhance the strength of adhesion. These may include roughening through physical means (e.g., sanding), chemical modification and fine etching (e.g., plasma treatment), and a combination of these treatments. When these treatments were applied to fused deposition modeling (FDM) 3D-printed components fabricated from polylactic acid (PLA), polylactic acid with wood flour additive (Wood-PLA), or acrylonitrile-butadiene-styrene (ABS) polymers, exposure to plasma resulted in a significant increase in surface wettability in all samples, and an improvement in the bond tensile shear strength of the polyvinyl acetate adhesive used to join these plastics to wood. Among the three treatment regimes, the combination treatment of plasma and sanding produced superior outcomes with respect to improving adhesion strength. Microscopic visualization of thus-treated surfaces showed the development of significant roughness after sanding, with some of this topography lost when the surface was subsequently exposed to plasma-induced ion bombardment [91].

In addition to understanding the strength of adhesion at the point of creating a composite structure, it is also important to study how the proposed surface treatments may affect the interlaminar crack propagation through opening, shearing, or their combination when the composite is subjected to mechanical load. Marino et al. studied the effect of plasma treatment on delamination in interlayer fiber hybrid composite laminates made of carbon/S-glass fiber epoxy and interleaved with oxygen plasma treated thin films of poly(acrylonitrile-butadiene-styrene) (ABS) and polystyrene (PS) [92] (Figure 10c,d). These thermoplastic interlayers were introduced to delay and/or suppress delamination through shearing in these hybrid composites and enhance their toughness. Plasma treatment enhanced wettability of the PS thermoplastic interlayers, positively contributing to the strength of bonding between the layers and the epoxy matrix, whereas the chemistry of ABS surfaces remained unchanged as a result of the plasma treatment. The delamination propagation was not substantially different between structures containing pristine and plasma-treated ABS interlayers, following a slow and symmetric path, with the stable and uniform plateau observed in the transition stage attributed to the reduced value of stress at which the delamination began to propagate. It has been suggested that the exposure of this thermoplastic resulted in material degradation that reduced its toughness and shear strength, rendering the treated material more susceptible to shear deformation and to plastic deformation that would blunt micro-cracks, thereby reducing the stress concentration in the proximity of the crack tip. This degradation of interlayer properties may be responsible for the observed pseudo-ductile failure of hybrid composites. In contrast, the same type of plasma treatment was able to introduce oxygen containing functional groups into PS, namely O-C=O or O–C–O–O, known to initiate the nucleophilic attacks on the epoxy rings of the composite material, resulting in a stable covalent bond and improved adhesion between the layers. This covalent bonding between the plasma-treated interlayer and the epoxy also provided another mechanism for the absorption of energy through crack deflection, and increasing the delamination stress and fracture toughness associated with shear delamination in the composite.

**Figure 11:**
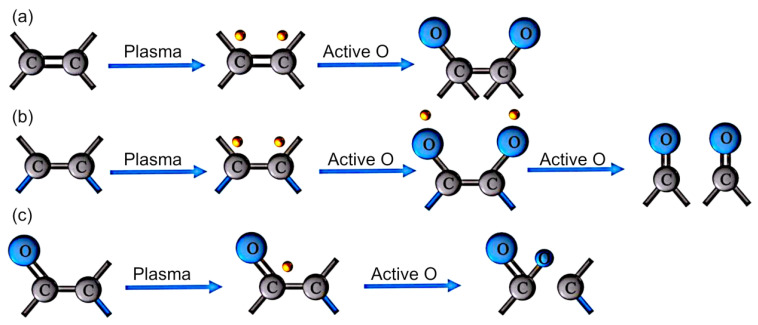
Possible mechanism of surface group oxidation by plasma treatment: (**a**) generation of C–O bonds, (**b**) generation of C=O bonds, (**c**) generation of O–C=O bonds. Reproduced from Lu et al. (2019) [30] under conditions of the CC BY license.

Lu et al. demonstrated the enhancement of the interfacial strength of carbon fiber/poly(ether ether ketone) hybrid composites by plasma treatment [30]. Fiber-reinforced thermoplastic composites such as hybrid composites based on carbon fibers and poly(ether ether ketone) (CF/PEEK) are considered as viable options to replace conventional prepreg systems for applications across automotive, aerospace, and other industries. These composites offer an attractive combination of flexibility and conformability as well as desirable operating performance including under high temperature. However, their fabrication remains a challenge because it is difficult for the polymer to infiltrate carbon fibers, and the melting viscosity of the polymer is high. Moreover, because the surface of the carbon fibers is smooth, chemically inactive, and hydrophobic, creating strong bonds between these surfaces and polymer fibers is not trivial, and unless dealt with, this issue can undermine the mechanical strength of the composite. In this study, both carbon and polymer fibers were exposed to radiofrequency (RF) plasma treatment using air, Ar, or Ar–air as processing gases. Plasma treatment activated the surface through chemical modification and surface roughening, resulting in the improved strength of interfacial interactions and greater fiber interlocking. Even a brief exposure of 1 min to Ar or air plasma was sufficient to attain a significant increase in the strength of the interfacial shear. Figure 11 illustrates possible mechanisms of surface group oxidation by plasma treatment: (a) generation of C–O bonds; (b) generation of C=O bonds; and (c) generation of O–C=O bonds. It should be noted that while plasma exposure was an efficient means to enrich the surfaces of fibers with oxygen moieties, it did not improve either the tensile strength or thermal properties of carbon and polymer fibers, respectively, even after the time of treatment was extended to 10 min.

The real-life applicability of plasma technologies in managing mechanical properties and adhesion of macroscopic objects rests with the ability to develop equipment that would be able to deliver plasma treatment at the necessary scale at a reasonable cost as well as the ability to integrate such a processing step into an existing workflow. Depending on the context, this may mean the development of portable processing units that could be used at the point of assembly (to avoid loss of desired chemistry over time), or the fabrication of sufficiently large atmospheric pressure plasma arrays that could be introduced within an existing processing line infrastructure at low cost.

## 6. Plasma for Layered and Hierarchical Structures of Polymers

Multi-layered structures of polymers and other materials is a very significant technology that allows for the design of very strong and light-weighted structures for various applications. Plasma could be an efficient method to ensure reliable, secure adhesion of metals, and other design materials to polymers.

**Figure 12:**
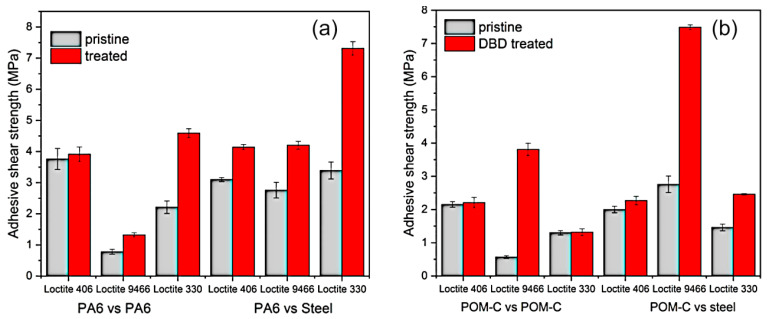
(**a**) Adhesive shear strength of pristine (grey) and plasma-treated (red) polymer/polymer and polymer/steel joints using various adhesives for PA6 (**a**) and POM-C (**b**). Reproduced from Károly et al. (2018) [93] under conditions of the CC BY license.

The application of a diffuse coplanar surface barrier discharge (DCSBD) technique for the improvement of adhesion properties of engineered polymers such as polyamide 6 and polyoxymethylene has been described by Károly et al. [93]. The authors demonstrated that exposure of these polymers to plasmas generated using air as a processing gas at an input power of 320 W improved the adhesion of these engineered polymers to other polymers as well as to steel joints, with cyanoacrylate (Loctite 406), an acrylic based adhesive (Loctite 3035) or a two-component epoxy adhesive (Loctite 9466) used to bond the surfaces together. PA6 experienced a more pronounced improvement in adhesion strength as a result of plasma treatment when compared to POM-C (Figure 12a,b). Where the adhesive delaminated from the surface of unmodified PA6, subsequent to plasma exposure, the adhesive would be removed from the surface of the steel rather than the polymer, confirming the treatment improved the polymer/adhesive interfacial adhesion. In the case of POM-C, there was not a significant difference in the nature of the delamination, with failure taking place primarily at the polymer/adhesive interface. It should be noted that greater values of shear stress had to be applied to initiate the failure.

**Figure 13:**
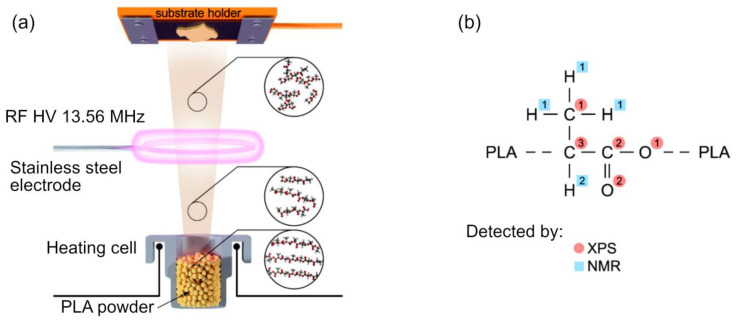
(**a**) Experimental setup used for PAVTD of PLA-like coatings and (**b**) chemical structure of conventional PLA with the assignment of C, O, and H that corresponds to different spectral peaks detectable by XP and NMR, respectively. Reproduced from Krtouš et al. (2021) [94] under conditions of the CC BY license.

**Figure 14:**
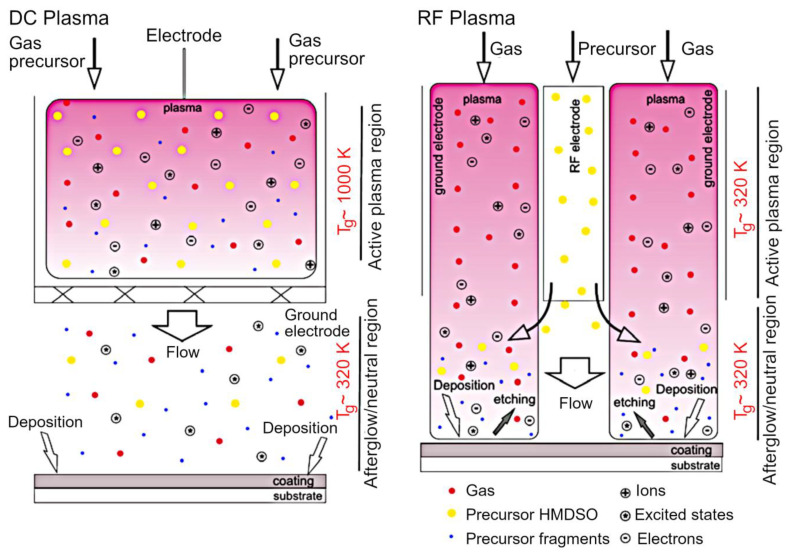
Schematic representation of the deposition process in DC and RF plasma jet. Films with lower fraction of carbon were fabricated using RF plasma, with the outcome related to the difference in the injection point of the source material. Reproduced from Kuchakova et al. (2020) [95] under conditions of the CC BY license.

Krtouš et al. demonstrated that the nature of the plasma-assisted vapor thermal deposition (PAVTD) offers numerous benefits for material processing, among them the ability to fabricate thin films that are compact and are characterized by a well-defined and controlled film thickness virtually on any type of substrate. Figure 13a,b show the experimental setup used for PAVTD of polylactic acid (PLA)-like coatings, and the chemical structure of conventional PLA. The primary advantage of this approach is that it has the potential to overcome one of the primary limitations of plasma polymers, namely the fact that under conventional plasma polymerization conditions, these polymers are built from small fragments of the original monomer molecules, resulting in a chemistry that is substantially different from that of polymer produced using conventional chemical pathways. While such fragmentation is not necessarily detrimental under all circumstances, for example, it may produce polymers with similar properties from monomers with slightly different chemistry, it is a limiting factor for cases where retention of original monomer chemistry is desired. In the approach described by Krtouš et al., traditional low molecular weight, volatile monomer units typically used in conventional plasma polymerization are replaced by high molecular weight precursors. In study, the source material comes in the form of polylactic acid granules. These granules are subjected to increased temperature under reduced pressure in an inert argon atmosphere to induce thermal polymer decomposition and generation of low- and high-molar mass fragments. These fragments can then undergo activation or fragmentation through the collisions with plasma species, initiating polymer formation. By tuning the heating regime and plasma parameters, it is possible to deposit polymers with properties that fall between traditional plasma and chemically-synthesized polymers, broadening the use of these polymers [94]. The deposition of organosilicon thin films by direct current and radio-frequency plasmas under atmospheric pressure conditions have been recently demonstrated by Kuchakova et al. [95]. As a source of polymer building blocks, hexamethyldisiloxane (HMDSO) was used. In the case of nitrogen DC plasma, substrates were mounted in the plasma afterglow, whereas in the case of argon RF plasma jets, substrates were exposed to the expanding plasma to produce materials with different surface topography and chemistry. Despite the differences in precursor injection, both types of films contained high levels of SiO_2_ of >98%, although polymers synthesized in the plasma afterglow were softer and had a greater level of roughness when compared to smooth polymers fabricated under a direct injection regime. Film failure through delamination and crack initiation and propagation was detected when organosilicon films were grown on polytetrafluoroethylene (PTFE) and polyetheretherketone (PEEK) substrates. Nevertheless, these two approaches show promise for the synthesis of SiO_x_ polymer coatings on a wide range of substrates, from polymers, to ceramics and metals. The scheme of the experimental setup is illustrated in Figure 14.

Plasma was also successfully used for the fabrication of other types of composites and multi-layer structures such as polymer/ZnO composites [96], calcium phosphate layers on thermally sensitive polymers [97], fibers for polymer composites [98], and titanium coatings on polymers [99].

For the development of complex structures, a combination of multiple plasma technologies within a single processing environment may be beneficial, as it may provide considerable savings in terms of time, energy, and cost of processing, and possibly greater efficiency and resolution. Baranov et al. [20] gave an example of one such ‘universal’ platform for plasma processing at low pressures, where the benefits of remaining within a single environment would deliver the most considerable savings with respect to time needed to bring the chamber to the needed level of vacuum multiple times. The proposed platform incorporated several plasma sources that could produce carbon and metallic plasmas, namely vacuum arc, RF, helicon and magnetron plasma sources, and a selection of magnetic coils to control the flux of energy and matter toward the surface, enhance efficiency through plasma confinement, and enhance control over species selectivity. This type of a platform could support any sequence of highly-selective etching of surface layers and nano-structuring, functionalization, and deposition of thin coatings or synthesis of nano- and micro-scale surface features. The resulting products can range from highly uniform surfaces to complex morphologies comprising hierarchical single- and multi-component architectures. While the development of such platforms is not without it challenges due to inherent limitations of existing technologies, the advantages of combining multiple sources may bring long-term significant benefits [20].

## 7. Future Trends

Despite the apparent simplicity of many of the plasma-enhanced experimental setups, the physical and chemical processes that take place in plasma bulk and in particular at the plasma–surface interfaces are numerous and dynamic, with a multitude of possible feedback loops that exist between these processes. For us to be able to take full advantage of the unique benefits offered by plasma-based technologies and processing environments, we need to have a comprehensive picture of these processes and interlinks. This holds true not only for material processing [100,101,102,103], where understanding and control of interfacial processes is critical, but also for other areas where plasmas are used [104,105,106,107,108,109]. The development of more sophisticated methods of diagnostics of interfacial and bulk processes, particularly under atmospheric pressure, open air conditions, and for the processing of materials with surface patterns of chemical or physical features, or any other type of heterogeneity, is essential to achieve progress in this field. The use of artificial intelligence and automation should facilitate large volume data collection while offering a greater degree of precision. To complement these experimental studies, modeling and simulation efforts should provide the much-needed backbone for process optimization and intelligent design of processes and materials for a given application. The significance of the feedback loops that may exist between processes and features, and their dynamic nature should be better investigated.

## Data Availability

Not applicable.

## References

[1] Rycroft M.J. (1986). Plasma—The fourth state of matter?. Nat. Cell Biol..

[2] Langmuir I. (1928). Oscillations in Ionized Gases. Proc. Natl. Acad. Sci. USA.

[3] Bazaka K., Bazaka O., Levchenko I., Xu S., Ivanova E.P., Keidar M., Ostrikov K.K. (2017). Plasma-potentiated small molecules—Possible alternative to antibiotics?. Nano Futur..

[4] Poh H.S., Lee M.C., Yap S.S., Teow S.Y., Bradley D.A. (2020). Potential use of plasma focus radiation sources in superficial cancer therapy. Jpn. J. Appl. Phys..

[5] Bazaka K., Levchenko I., Lim J.W.M., Baranov O., Corbella C., Xu S., Keidar M., Keidar M. (2019). MoS2-based nanostructures: Synthesis and applications in medicine. J. Phys. D Appl. Phys..

[6] Pan S., Zhang S., Chen H. (2020). Low temperature plasma promotes the healing of chronic wounds in diabetic mice. J. Phys. D Appl. Phys..

[7] Chen Z., Zhang S., Levchenko I., Beilis I.I., Keidar M. (2017). In vitro Demonstration of Cancer Inhibiting Properties from Stratified Self-Organized Plasma-Liquid Interface. Sci. Rep..

[8] Bazaka K., Jacob M., Ostrikov K.K. (2016). Sustainable Life Cycles of Natural-Precursor-Derived Nanocarbons. Chem. Rev..

[9] Tamilselvi R., Ramesh M., Lekshmi G., Bazaka O., Levchenko I., Bazaka K., Mandhakini M. (2020). Graphene oxide-based supercapacitors from agricultural wastes: A step to mass production of highly efficient electrodes for electrical transportation systems. Renew. Energy.

[10] Alancherry S., Jacob M., Prasad K., Joseph J., Bazaka O., Neupane R., Varghese O., Baranov O., Xu S., Levchenko I. (2020). Tuning and fine morphology control of natural resource-derived vertical graphene. Carbon.

[11] Zhou H.P., Ye X., Huang W., Wu M.Q., Mao L.N., Yu B., Xu S., Levchenko I., Bazaka K. (2019). Wearable, Flexible, Disposable Plasma-Reduced Graphene Oxide Stress Sensors for Monitoring Activities in Austere Environments. ACS Appl. Mater. Interfaces.

[12] Levchenko I., Bazaka K., Belmonte T., Keidar M., Xu S. (2018). Advanced Materials for Next-Generation Spacecraft. Adv. Mater..

[13] Levchenko I., Xu S., Wu Y.-L., Bazaka K. (2020). Hopes and concerns for astronomy of satellite constellations. Nat. Astron..

[14] Levchenko I., Baranov O., Fang J., Cherkun O., Xu S., Bazaka K. (2021). Focusing plasma jets to achieve high current density: Feasibility and opportunities for applications in debris removal and space exploration. Aerosp. Sci. Technol..

[15] Levchenko I., Keidar M., Cantrell J., Wu Y.-L., Kuninaka H., Bazaka K., Xu S. (2018). Explore space using swarms of tiny satellites. Nat. Cell Biol..

[16] Lemmer K. (2017). Propulsion for CubeSats. Acta Astronaut..

[17] Lim J.W.M., Levchenko I., Huang S., Xu L., Sim R., Yee J.S., Potrivitu G., Sun Y., Bazaka K., Wen X. (2019). Plasma parameters and discharge characteristics of lab-based krypton-propelled miniaturized Hall thruster. Plasma Sources Sci. Technol..

[18] Levchenko I., Bazaka K., Mazouffre S., Xu S. (2018). Prospects and physical mechanisms for photonic space propulsion. Nat. Photon..

[19] Charles C. (2009). Plasmas for spacecraft propulsion. J. Phys. D Appl. Phys..

[20] Baranov O., Xu S., Ostrikov K., Wang B.B., Cvelbar U., Bazaka K., Levchenko I. (2018). Towards universal plasma-enabled platform for the advanced nanofabrication: Plasma physics level approach. Rev. Mod. Plasma Phys..

[21] Levchenko I., Bazaka K., Ding Y., Raitses Y., Mazouffre S., Henning T., Klar P.J., Shinohara S., Schein J., Garrigues L. (2018). Space micropropulsion systems for Cubesats and small satellites: From proximate targets to furthermost frontiers. Appl. Phys. Rev..

[22] Mazouffre S. (2016). Electric propulsion for satellites and spacecraft: Established technologies and novel approaches. Plasma Sources Sci. Technol..

[23] Baranov O., Levchenko I., Xu S., Wang X.G., Zhou H.P., Bazaka K. (2019). Direct current arc plasma thrusters for space applications: Basic physics, design and perspectives. Rev. Mod. Plasma Phys..

[24] Karadag B., Cho S., Funaki I. (2018). Thrust performance, propellant ionization, and thruster erosion of an external discharge plasma thruster. J. Appl. Phys..

[25] Levchenko I., Ostrikov K.K., Keidar M., Vladimirov S. (2007). Angular distribution of carbon ion flux in a nanotube array during the plasma process by the Monte Carlo technique. Phys. Plasmas.

[26] Baranov O., Bazaka K., Kersten H., Keidar M., Cvelbar U., Xu S., Levchenko I. (2017). Plasma under control: Advanced solutions and perspectives for plasma flux management in material treatment and nanosynthesis. Appl. Phys. Rev..

[27] Levchenko I., Romanov M., Korobov M. (2004). Current–voltage characteristics of a substrate in a crossed E×B field system exposed to plasma flux from vacuum arc plasma sources. Surf. Coatings Technol..

[28] Woller K., Whyte D., Wright G. (2017). Impact of helium ion energy modulation on tungsten surface morphology and nano-tendril growth. Nucl. Fusion.

[29] Marvi Z., Ostrikov K., Levchenko I., Xu S., Foroutan G. (2017). Plasma-deposited hydrogenated amorphous silicon films: Multiscale modelling reveals key processes. RSC Adv..

[30] Lu C., Qiu S., Lu X., Wang J., Xiao L., Zheng T., Wang X., Zhang D. (2019). Enhancing the Interfacial Strength of Carbon Fiber/Poly(ether ether ketone) Hybrid Composites by Plasma Treatments. Polymers.

[31] Anders A., Anders S. (1995). The working principle of the hollow-anode plasma source. Plasma Sources Sci. Technol..

[32] Levchenko I., Korobov M., Romanov M., Keidar M. (2004). Ion current distribution on a substrate during nanostructure formation. J. Phys. D Appl. Phys..

[33] Gruart M., Feldberg N., Gayral B., Bougerol C., Pouget S., Bellet-Amalric E., Garro N., Cros A., Okuno H., Daudin B. (2019). Impact of kinetics on the growth of GaN on graphene by plasma-assisted molecular beam epitaxy. Nanotechnology.

[34] Baranov O., Levchenko I., Bell J.M., Lim J.W.M., Huang S., Xu L., Wang B.B., Aussems D.U.B., Xu S., Bazaka K. (2018). From nanometre to millimetre: A range of capabilities for plasma-enabled surface functionalization and nanostructuring. Mater. Horizons.

[35] Levchenko I., Ostrikov K., Keidar M., Xu S.L.Y. (2005). Microscopic ion fluxes in plasma-aided nanofabrication of ordered carbon nanotip structures. J. Appl. Phys..

[36] Levchenko I., Bazaka K., Baranov O., Sankaran R.M., Nominé A., Belmonte T., Xu S. (2018). Lightning under water: Diverse reactive environments and evidence of synergistic effects for material treatment and activation. Appl. Phys. Rev..

[37] Ivanova E., Bazaka K., Crawford R. (2014). New Functional Biomaterials for Medicine and Healthcare. New Functional Biomaterials for Medicine and Healthcare.

[38] Turicek J., Ratts N., Kaltchev M., Masoud N. (2021). Surface Treatment of Ultra-High Molecular Weight Polyethylene (UHMWPE) by Cold Atmospheric Plasma (CAP) for Biocompatibility Enhancement. Appl. Sci..

[39] Totaro K.A., Liao X., Bhattacharya K., Finneman J.I., Sperry J.B., Massa M.A., Thorn J., Ho S.V., Pentelute B.L. (2016). Systematic Investigation of EDC/sNHS-Mediated Bioconjugation Reactions for Carboxylated Peptide Substrates. Bioconjugate Chem..

[40] Wieland F., Bruch R., Bergmann M., Partel S., Urban G.A., Dincer C. (2020). Enhanced Protein Immobilization on Polymers—A Plasma Surface Activation Study. Polymers.

[41] Miroshnichenko S., Timofeeva V., Permyakova E., Ershov S., Kiryukhantsev-Korneev P., Dvořaková E., Shtansky D.V., Zajíčková L., Solovieva A., Manakhov A. (2019). Plasma-Coated Polycaprolactone Nanofibers with Covalently Bonded Platelet-Rich Plasma Enhance Adhesion and Growth of Human Fibroblasts. Nanomaterials.

[42] Chang C.-K., Wang H.-M.D., Lan J.C.-W. (2018). Investigation and Characterization of Plasma-Treated Poly(3-hydroxybutyrate) and Poly(3-hydroxybutyrate-co-3-hydroxyvalerate) Biopolymers for an In Vitro Cellular Study of Mouse Adipose-Derived Stem Cells. Polymers.

[43] Jeong Y.-W., Jung S., Han J.J., Park H.-J., Kim R.Y., Kim B.-H., Kook M.-S. (2019). Effectiveness of Surface Treatment with Amine Plasma for Improving the Biocompatibility of Maxillofacial Plates. Materials.

[44] Stahel P., Mazánková V., Tomečková K., Matoušková P., Brablec A., Prokeš L., Jurmanová J., Buršíková V., Přibyl R., Lehocký M. (2019). Atmospheric Pressure Plasma Polymerized Oxazoline-Based Thin Films—Antibacterial Properties and Cytocompatibility Performance. Polymers.

[45] Nicol M.J., Brubaker T.R., Honish B.J., Simmons A.N., Kazemi A., Geissel M.A., Whalen C.T., Siedlecki C.A., Bilén S.G., Knecht S.D. (2020). Antibacterial effects of low-temperature plasma generated by atmospheric-pressure plasma jet are mediated by reactive oxygen species. Sci. Rep..

[46] Bazaka K., Jacob M., Truong V.K., Wang F., Pushpamali W.A.A., Wang J.Y., Ellis A.V., Berndt C.C., Crawford R., Ivanova E.P. (2010). Plasma-Enhanced Synthesis of Bioactive Polymeric Coatings from Monoterpene Alcohols: A Combined Experimental and Theoretical Study. Biomacromolecules.

[47] Bazaka K., Jacob M.V., Truong V.K., Crawford R.J., Ivanova E.P. (2011). The Effect of Polyterpenol Thin Film Surfaces on Bacterial Viability and Adhesion. Polymers.

[48] Al-Jumaili A., Bazaka K., Jacob M.V. (2017). Retention of Antibacterial Activity in Geranium Plasma Polymer Thin Films. Nanomater..

[49] Bazaka K., Destefani R., Jacob M.V. (2016). Plant-derived cis-β-ocimene as a precursor for biocompatible, transparent, thermally-stable dielectric and encapsulating layers for organic electronics. Sci. Rep..

[50] Al-Jumaili A., Mulvey P., Kumar A., Prasad K., Bazaka K., Warner J., Jacob M.V. (2019). Eco-friendly nanocomposites derived from geranium oil and zinc oxide in one step approach. Sci. Rep..

[51] Kumar A., Grant D.S., Bazaka K., Jacob M.V. (2018). Tailoring terpenoid plasma polymer properties by controlling the substrate temperature during PECVD. J. Appl. Polym. Sci..

[52] Kumar A., Al-Jumaili A., Prasad K., Bazaka K., Mulvey P., Warner J., Jacob M.V. (2020). Pulse Plasma Deposition of Terpinen-4-ol: An Insight into Polymerization Mechanism and Enhanced Antibacterial Response of Developed Thin Films. Plasma Chem. Plasma Process..

[53] Ahmad J., Bazaka K., Whittle J.D., Michelmore A., Jacob M.V. (2015). Structural Characterization of γ-Terpinene Thin Films Using Mass Spectroscopy and X-Ray Photoelectron Spectroscopy. Plasma Process. Polym..

[54] Bazaka K., Ahmad J., Oelgemöller M., Uddin A., Jacob M. (2017). Photostability of plasma polymerized γ-terpinene thin films for encapsulation of OPV. Sci. Rep..

[55] Sakudo A., Yagyu Y., Onodera T. (2019). Disinfection and Sterilization Using Plasma Technology: Fundamentals and Future Perspectives for Biological Applications. Int. J. Mol. Sci..

[56] Bazaka K., Jacob M.V., Bowden B.F. (2011). Optical and chemical properties of polyterpenol thin films deposited via plasma-enhanced chemical vapor deposition. J. Mater. Res..

[57] Grant D.S., Bazaka K., Davies J.B., Banos C., Jacob M.V. (2017). Organic bioelectronic plasma polymerised polyterpenol thin films: Preservation of properties relevant to biomedical and organic electronic applications following exposure to sterilising doses of gamma radiation. J. Mater. Sci. Mater. Electron..

[58] Slepička P., Rimpelová S., Kasálková N.S., Fajstavr D., Sajdl P., Kolská Z., Švorčík V. (2021). Antibacterial Properties of Plasma-Activated Perfluorinated Substrates with Silver Nanoclusters Deposition. Nanomaterials.

[59] Bazaka K., Ketheesan N., Jacob M.V. (2014). Polymer encapsulation of magnesium to control biodegradability and biocompatibility. J. Nanosci. Nanotechnol..

[60] Scally L., Gulan M., Weigang L., Cullen P.J., Milosavljevic V. (2018). Significance of a Non-Thermal Plasma Treatment on LDPE Biodegradation with Pseudomonas Aeruginosa. Materials.

[61] Kumar A., Mills S., Bazaka K., Bajema N., Atkinson I., Jacob M.V. (2018). Biodegradable optically transparent terpinen-4-ol thin films for marine antifouling applications. Surf. Coatings Technol..

[62] Cavallaro A.A., MacGregor-Ramiasa M.N., Vasilev K. (2016). Antibiofouling Properties of Plasma-Deposited Oxazoline-Based Thin Films. ACS Appl. Mater. Interfaces.

[63] Ostrikov K., MacGregor-Ramiasa M., Cavallaro A., Ostrikov K.K., Vasilev K. (2016). Bactericidal effects of plasma-modified surface chemistry of silicon nanograss. J. Phys. D Appl. Phys..

[64] Al-Bataineh S.A., Cavallaro A.A., Michelmore A., Macgregor M.N., Whittle J.D., Vasilev K. (2019). Deposition of 2-oxazoline-based plasma polymer coatings using atmospheric pressure helium plasma jet. Plasma Process. Polym..

[65] MacGregor M., Sinha U., Visalakshan R.M., Cavallaro A., Vasilev K. (2019). Preserving the reactivity of coatings plasma deposited from oxazoline precursors—An in depth study. Plasma Process. Polym..

[66] Ramiasa M.N., Cavallaro A.A., Mierczynska A., Christo S.N., Gleadle J.M., Hayball J., Vasilev K. (2015). Plasma polymerised polyoxazoline thin films for biomedical applications. Chem. Commun..

[67] Macgregor-Ramiasa M.N., Cavallaro A.A., Vasilev K. (2015). Properties and reactivity of polyoxazoline plasma polymer films. J. Mater. Chem. B.

[68] Chan K.V., Asadian M., Onyshchenko I., Declercq H., Morent R., De Geyter N. (2019). Biocompatibility of Cyclopropylamine-Based Plasma Polymers Deposited at Sub-Atmospheric Pressure on Poly (ε-caprolactone) Nanofiber Meshes. Nanomaterials.

[69] Fleming G., Aveyard J., Fothergill J.L., McBride F., Raval R., D’Sa R.A. (2017). Nitric Oxide Releasing Polymeric Coatings for the Prevention of Biofilm Formation. Polymers.

[70] Holvoet S., Chevallier P., Turgeon S., Mantovani D. (2010). Toward High-Performance Coatings for Biomedical Devices: Study on Plasma-Deposited Fluorocarbon Films and Ageing in PBS. Materials.

[71] Popelka A., Novak I., Lehocký M., Chodák I., Sedliačik J., Gajtanska M., Sedliačiková M., Vesel A., Junkar I., Kleinová A. (2012). Anti-bacterial Treatment of Polyethylene by Cold Plasma for Medical Purposes. Molecules.

[72] Levchenko I., Xu S., Cherkun O., Baranov O., Bazaka K. (2021). Plasma meets metamatertials: Three ways to advance space micropropulsion systems. Adv. Phys. X.

[73] Panaitescu D.M., Vizireanu S., Stoian S.A., Nicolae C.-A., Gabor A.R., Damian C.M., Trusca R., Carpen L.G., Dinescu G. (2020). Poly(3-hydroxybutyrate) Modified by Plasma and TEMPO-Oxidized Celluloses. Polymers.

[74] Di Mundo R., Bottiglione F., Notarnicola M., Palumbo F., Pascazio G. (2017). Plasma-Textured Teflon: Repulsion in Air of Water Droplets and Drag Reduction Underwater. Biomimetics.

[75] Pulyalina A., Rostovtseva V., Faykov I., Tataurov M., Dubovenko R., Shugurov S. (2021). Development of Novel Polyamide-Imide/DES Composites and Their Application for Pervaporation and Gas Separation. Molecules.

[76] Liang C.Z., Chung T.-S., Lai J.-Y. (2019). A review of polymeric composite membranes for gas separation and energy production. Prog. Polym. Sci..

[77] Zarshenas K., Raisi A., Aroujalian A. (2015). Surface modification of polyamide composite membranes by corona air plasma for gas separation applications. RSC Adv..

[78] Nagasawa H., Yamamoto Y., Tsuda N., Kanezashi M., Yoshioka T., Tsuru T. (2017). Atmospheric-pressure plasma-enhanced chemical vapor deposition of microporous silica membranes for gas separation. J. Membr. Sci..

[79] Wang J., Chen X., Reis R., Chen Z., Milne N., Winther-Jensen B., Kong L., Dumée L.F. (2018). Plasma Modification and Synthesis of Membrane Materials—A Mechanistic Review. Membranes.

[80] Laurano R., Boffito M., Torchio A., Cassino C., Chiono V., Ciardelli G. (2019). Plasma Treatment of Polymer Powder as an Effective Tool to Functionalize Polymers: Case Study Application on an Amphiphilic Polyurethane. Polymers.

[81] Stortini A.M., Fabris S., Saorin G., Falzacappa E.V., Moretto L.M., Ugo P. (2019). Plasma Activation of Copper Nanowires Arrays for Electrocatalytic Sensing of Nitrate in Food and Water. Nanomaterials.

[82] Sauerbier P., Köhler R., Renner G., Militz H. (2020). Surface Activation of Polylactic Acid-Based Wood-Plastic Composite by Atmospheric Pressure Plasma Treatment. Materials.

[83] Kaynak A., Mehmood T., Dai X.J., Magniez K., Kouzani A. (2013). Study of Radio Frequency Plasma Treatment of PVDF Film Using Ar, O_2_ and (Ar + O_2_) Gases for Improved Polypyrrole Adhesion. Materials.

[84] Iqbal M., Dinh D.K., Abbas Q., Imran M., Sattar H., Ahmad A.U. (2019). Controlled Surface Wettability by Plasma Polymer Surface Modification. Surfaces.

[85] Wang K., Tan H., Lin Y., Diono W., Zhao Y., Goto M. (2020). Direct current gas–liquid phase pulsed plasma polymerization of polypyrrole under atmospheric pressure. Plasma Process. Polym..

[86] Jalaber V., Del Frari D., De Winter J., Mehennaoui K., Planchon S., Choquet P., Detrembleur C., Moreno-Couranjou M. (2019). Atmospheric Aerosol Assisted Pulsed Plasma Polymerization: An Environmentally Friendly Technique for Tunable Catechol-Bearing Thin Films. Front. Chem..

[87] Vesel A., Zaplotnik R., Primc G., Mozetič M. (2020). Evolution of the Surface Wettability of PET Polymer upon Treatment with an Atmospheric-Pressure Plasma Jet. Polymers.

[88] Licciardello M., Ciardelli G., Tonda-Turo C. (2021). Biocompatible Electrospun Polycaprolactone-Polyaniline Scaffold Treated with Atmospheric Plasma to Improve Hydrophilicity. Bioengineering.

[89] Hunke H., Soin N., Shah T.H., Krämer E., Pascual A., Karuna M.S.L., Siores E. (2015). Low-Pressure H2, NH3 Microwave Plasma Treatment of Polytetrafluoroethylene (PTFE) Powders: Chemical, Thermal and Wettability Analysis. Materials.

[90] Yáñez-Pacios A.J., Martin-Martinez J.M. (2018). Comparative Adhesion, Ageing Resistance, and Surface Properties of Wood Plastic Composite Treated with Low Pressure Plasma and Atmospheric Pressure Plasma Jet. Polymers.

[91] Kariž M., Tomec D., Dahle S., Kuzman M., Šernek M., Žigon J. (2021). Effect of Sanding and Plasma Treatment of 3D-Printed Parts on Bonding to Wood with PVAc Adhesive. Polymers.

[92] Marino S.G., Mayer F., Bismarck A., Czél G. (2020). Effect of Plasma-Treatment of Interleaved Thermoplastic Films on Delamination in Interlayer Fibre Hybrid Composite Laminates. Polymers.

[93] Károly Z., Kalácska G., Zsidai L., Mohai M., Klébert S. (2018). Improvement of Adhesion Properties of Polyamide 6 and Polyoxymethylene-Copolymer by Atmospheric Cold Plasma Treatment. Polymers.

[94] Krtouš Z., Hanyková L., Krakovský I., Nikitin D., Pleskunov P., Kylián O., Sedlaříková J., Kousal J. (2021). Structure of Plasma (re)Polymerized Polylactic Acid Films Fabricated by Plasma-Assisted Vapour Thermal Deposition. Materials.

[95] Kuchakova I., Ionita M.D., Ionita E.-R., Lazea-Stoyanova A., Brajnicov S., Mitu B., Dinescu G., De Vrieze M., Cvelbar U., Zille A. (2020). Atmospheric Pressure Plasma Deposition of Organosilicon Thin Films by Direct Current and Radio-frequency Plasma Jets. Materials.

[96] Al-Jumaili A., Kumar A., Bazaka K., Jacob M.V. (2019). Electrically Insulating Plasma Polymer/ZnO Composite Films. Materials.

[97] Groza A., Dreghici D.B., Ganciu M. (2019). Calcium Phosphate Layers Deposited on Thermal Sensitive Polymer Substrates in Radio Frequency Magnetron Plasma Discharge. Coatings.

[98] Hamad S.F., Stehling N., Hayes S.A., Foreman J.P., Rodenburg C. (2019). Exploiting Plasma Exposed, Natural Surface Nanostructures in Ramie Fibers for Polymer Composite Applications. Materials.

[99] Wypych A., Siwak P., Andrzejewski D., Jakubowicz J. (2018). Titanium Plasma-Sprayed Coatings on Polymers for Hard Tissue Applications. Materials.

[100] Uchida K., Tanaka N. (2019). Nanoscale, low-energy molecular sensors for health care and environmental monitoring. AAPPS Bull..

[101] Levchenko I., Xu S., Teel G., Mariotti D., Walker M., Keidar M. (2018). Recent progress and perspectives of space electric propulsion systems based on smart nanomaterials. Nat. Commun..

[102] Singhal N., Levchenko I., Huang S., Xu L., Potrivitu G.-C., Cherkun O., Fang J., Bazaka K., Xu S. (2019). 3D-Printed Multilayered Reinforced Material System for Gas Supply in CubeSats and Small Satellites. Adv. Eng. Mater..

[103] Levchenko I., Bazaka K., Keidar M., Xu S., Fang J. (2018). Hierarchical Multicomponent Inorganic Metamaterials: Intrinsically Driven Self-Assembly at the Nanoscale. Adv. Mater..

[104] Levchenko I., Xu S., Mazouffre S., Lev D., Pedrini D., Goebel D., Garrigues L., Taccogna F., Bazaka K. (2020). Perspectives, frontiers, and new horizons for plasma-based space electric propulsion. Phys. Plasmas.

[105] Takase K., Takahashi K., Takao Y. (2018). Effects of neutral distribution and external magnetic field on plasma momentum in electrodeless plasma thrusters. Phys. Plasmas.

[106] Lim J.W.M., Huang S.Y., Xu L., Yee J.S., Sim R.Z., Zhang Z.L., Levchenko I., Xu S. (2018). Automated integrated robotic systems for diagnostics and test of electric and micropropulsion thrusters. IEEE Trans. Plasma Sci..

[107] Levchenko I., Romanov M., Keidar M., Beilis I.I. (2004). Stable plasma configurations in a cylindrical magnetron discharge. Appl. Phys. Lett..

[108] Ding Y., Wang L., Fan H., Li H., Xu W., Wei L., Li P., Yu D. (2019). Simulation research on magnetic pole erosion of Hall thrusters. Phys. Plasmas.

[109] Levchenko I., Xu S., Mazouffre S., Keidar M., Bazaka K. (2018). Mars Colonization: Beyond Getting There. Glob. Chall..

